# SARS-CoV-2 detection methods: A comprehensive review

**DOI:** 10.1016/j.sjbs.2022.103465

**Published:** 2022-09-27

**Authors:** Galyah Alhamid, Huseyin Tombuloglu, Ali A. Rabaan, Ebtesam Al-Suhaimi

**Affiliations:** a Department of Genetics Research, Institute for Research and Medical Consultations (IRMC), Imam Abdulrahman Bin Faisal University, P.O. Box 1982, Dammam 31441, Saudi Arabia; b Biotechnology Master Program, Imam Abdulrahman bin Faisal University, Saudi Arabia; c Molecular Diagnostic Laboratory, Johns Hopkins Aramco Healthcare, Dhahran, Saudi Arabia; d Department of Biology, College of Science and Institute of Research and Medical Consultations (IRMC), Imam Abdulrahman Bin Faisal University, 31441 Dammam, Saudi Arabia

**Keywords:** SARS-CoV-2, COVID-19, Detection methods, RT-qPCR, Specimens, Serology, Variants

## Abstract

The ongoing novel COVID-19 has remained the center of attention, since its declaration as a pandemic in March 2020, due to its rapid and uncontrollable worldwide spread. Diagnostic tests are the first line of defense against the transmission of this infectious disease among individuals, with reverse-transcription quantitative polymerase chain reaction (RT-qPCR) being the approved gold standard for showing high sensitivity and specificity in detecting SARS-CoV-2. However, alternative tests are being invested due to the global demand for facilities, reagents, and healthcare workers needed for rapid population-based testing. Also, the rapid evolution of the viral genome and the emergence of new variants necessitates updating the existing methods. Scientists are aiming to improve tests to be affordable, simple, fast, and at the same time accurate, and efficient, as well as friendly user testing. The current diagnostic methods are either molecular-based that detect nucleic acids abundance, like RT-qPCR and reverse-transcription loop-mediated isothermal amplification (RT-LAMP); or immunologically based that detect the presence of antigens or antibodies in patients’ specimens, like enzyme-linked immunosorbent assay (ELISA), lateral flow assay (LFA), chemiluminescent immunoassay (CLIA), and neutralization assay. In addition to these strategies, sensor-based or CRISPR applications are promising tools for the rapid detection of SARS-CoV-2. This review summarizes the most recent updates on the SARS-CoV-2 detection methods with their limitations. It will guide researchers, epidemiologists, and clinicians in identifying a more rapid, reliable, and sensitive method of diagnosing SARS-CoV-2 including the most recent variant of concern Omicron.

## 1 Introduction

The Severe Acute Respiratory Syndrome Coronavirus 2 (SARS-CoV-2) is a positive single-stranded enveloped RNA virus that caused the novel coronavirus disease 2019 (COVID-19) pandemic. Around 604 million people were infected, with over six million deaths worldwide as of August 2022, according to World Health Organization (WHO) (WHO, 2022, COVID-19 Map , 2022). This disease is highly contagious and can spread rapidly to the respiratory tract through close contact with infected individuals via talking, coughing, and sneezing. Most patients suffer mild to moderate symptoms ranging from fever, cough, and fatigue to pneumonia, while few require hospitalization and mechanical ventilators. High-risk groups—elders over 65 years old, obese, patients with chronic diseases, or impaired immune system—may develop serious complications that lead to septic shock, multiorgan failure, and death. On the other hand, some patients develop no symptoms (known to be asymptomatic) but can still transmit the disease to others (Wang et al., 2020, Zhou et al., 2020, Khan et al., 2020). Yet, studies reported no significant difference in the viral loads between asymptomatic, pre-symptomatic, and symptomatic patients, as reviewed by Walsh et al. and Zuin et al. (Walsh et al., 2020;, Zuin et al., 2021). However, other studies reported a positive association between high viral loads and the severity of symptoms (Walsh et al., 2020). This virus has overwhelmingly outstripped the SARS and MERS outbreaks, both in terms of the number of infected people and the geographical spread of the epidemic (Hu et al., 2021). Moreover, this crisis, which emerged in late 2019, continues to affect today and poses an extraordinary threat to public health and socioeconomics on a global scale. Unpredictable effects of genetic variations (mutations) on functional proteins can worsen this situation in the near future by altering the character of the virus in terms of its rate of transmission and infectiousness. Also, asymptomatic individuals are estimated to spread the disease 75 % more than symptomatic cases, potentially making the viral spread uncontrolled (Ferguson and Laydon, 2020).

To keep this pandemic at bay, expanded and rapid testing is crucial to limit the uncontrolled spreading of the disease. Reverse transcription-quantitative polymerase chain reaction (RT-qPCR) was declared the gold standard by WHO. However, this method requires bulky and expensive instrumentation in laboratory settings operated only by experienced personnel, which is quite challenging to catch up to the number of tests per day compared to the resources available. Alternative methods are needed for on-site, simple, and fast testing to cover broader populations in regions with limited resources or resolve the global demand for reagents and diagnostic equipment. Therefore, researchers are continuing the studies to develop fast, affordable, and practical solutions to detect the novel SARS-CoV-2. In this review, we summarize recent developments in sampling and diagnostic methods to identify a more rapid, reliable, and sensitive method of diagnosing SARS-CoV-2 infections. Based on the recently published findings, the genome organization and up-to-date emerging variants of SARS-CoV-2 and clinical applications such as sample selection, serological, and molecular techniques for virus identification are comprehensively reviewed.

## 2 Genome organization of SARS-CoV-2 and emerging variants

**Fig. 1:**
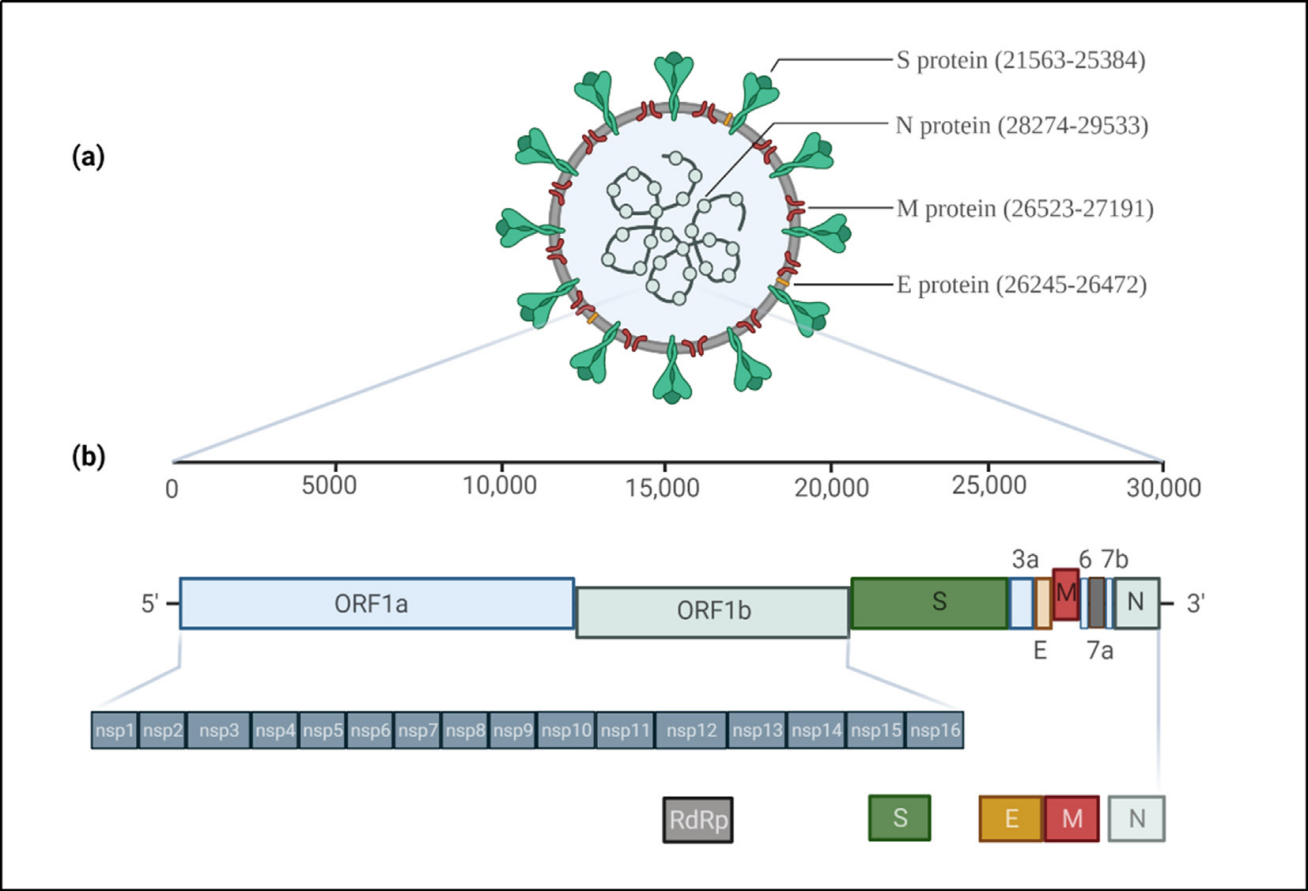
**(a)** SARS-CoV-2 structural proteins and their genomic locations. **(b)** The genome organization of the viral genome residing nonstructural proteins (*nsp*) and structural protein-encoding genes (*S*, *E*, *M*, and *N*). nsp12, translated by open reading frame (ORF1ab), encodes for RNA-dependent RNA polymerase.

Coronaviruses are known to be originated from animals. Some of them can co-exist with humans (human coronaviruses) as endemic infections and low pathogenic (HCoV-229E, HCoV-NL63, HCoV-OC43, HCoV-HKU1), while others such as severe acute respiratory syndrome coronavirus (SARS-CoV), SARS-CoV-2, and Middle East respiratory syndrome coronavirus (MERS-CoV) have evolved to cause severe diseases in humans with case-fatality rates of 10.9 %, 2.1 %, and 34.3 %, respectively (Singh et al., 2021). Together with SARS-CoV and MERS-CoV, SARS-CoV-2 belongs to the genus *Betacoronavirus*, the *Coronaviridae* family with great genetic diversity (Wang et al., 2020). With a size of 29.9 kb, the SARS-CoV-2 genome consists of open reading frames (ORF) that encode ORF1ab polyproteins at 5‘ end; and regions that encode nucleocapsid (N), membrane (M), envelope (E), and spike (S) structural proteins at 3‘ end. The S protein is composed of the S1 subunit, which contains the receptor-binding domain (RBD) that attaches to the angiotensin-converting enzyme 2 (ACE2) receptor on the host’s epithelial cells; and the S2 subunit, which mediates viral fusion and entry to host cells. Also, the SARS-CoV-2 genome has 16 nonstructural proteins (nsp), in which *nsp12* encodes RNA-dependent RNA polymerase (RdRp). This enzyme catalyzes the RNA synthesis from an RNA template, thereby responsible for viral replication (Wang et al., 2020, Zhou et al., 2020). Fig. 1 shows the whole genome structure of SARS-CoV-2 and its associated proteins with the genomic positions.

Like most viruses, SARS-CoV-2 underwent genetic mutations, or variations, that emerged from the original strain “L” from Wuhan, China, in late December 2019. The nucleotide mutation rate of the SARS-CoV-2 genome has been estimated as 6.677 × 10^−4^ substitution per site per year (Wang et al., 2022), which decrease the efficacy of diagnosis techniques, as shown by Penarrubia et al. (2020). The emergence of new variants and the high mutation rate of the viral genome require updating existing diagnostic tests. Since the start of the pandemic, WHO has been tracking and documenting the genetic lineages that pose clear evidence of risk to global public health via their increased transmissibility, infectivity, or decrease in the effectiveness of diagnostic tools, therapies, and vaccines. WHO describes lineages with these characteristics as the variants of concern (VOC). On the other hand, other SARS-CoV-2 variants with genetic mutations that have uncertain evidence globally in increasing transmissibility, the severity of the disease, and impacting immunity are referred to as variants of interest (VOI). Currently, there are no circulating VOI as of August 2022 (WHO, 2022). Table 1 shows the labels for the global SARS-CoV-2 variants and their scientific nomenclature, mutations, and earliest documented emergence date and origin, according to WHO and the center for disease control and prevention (CDC), with Omicron being the dominant VOC. Other variants previously identified as VOC are now downgraded to variants being monitored (VBM). Whole genome sequencing of the Omicron variant revealed over 30 amino acid mutations in the *S* gene, the majority of which are in the RBD. As described by (Venkatakrishnan et al., 2021), the Omicron genome acquired a unique insertion having three amino acids in size (ins214EPE), which has not been previously observed in any SARS-CoV-2 lineage. It is hypothesized that such insertion in the genome could be possible by a template switching mechanism. In this model, a genomic matter exchange in between the host genome (human) and viral genome (SARS-CoV-2) or viral genome (SARS-CoV-2) and another viral genome (such as Human coronavirus 229E (HCoV-229E)) is possible. With this variant, a higher reinfection risk was reported compared to others due to its increased ability to evade hosts’ immunity (WHO, 2022, CDC, 2022).

**Table 1:** SARS-CoV-2 VOC and VBM.

	Variant	Date of emergence	Mutations	Origin	Incidence	Attributes
**VOC**						
Omicron	B.1.1.529	November 2021	R346K L452X F486V	South Africa	> 99 %	Increased transmissibility and reinfection rate. Substantially reduced mortality and hospitalization.
**VBM**						
Alpha	B.1.1.7	September 2020	S484K S452R	United Kingdom	< 1 %	Increased transmissibility, hospitalization, and mortality compared to wild type.
Beta	B.1.351	May 2020	K417N, E484K, N501Y, D614G, A701V	South Africa	< 1 %	Increased transmissibility.
Gamma	P.1	November 2020	K417T, E484K, N501Y, D614G, H655Y	Brazil	< 1 %	Increased transmissibility.
Delta	B.1.617.2	October 2020	L452R, T478K, D614G, P681R	India	< 1 %	Significantly increased transmissibility, hospitalization, and mortality rates.

Abbreviations: VOC, variants of concern; VBM, variants being monitored.

## 3 Types of specimens

SARS-CoV-2 can be detected in various specimens, including swabs (nasopharyngeal, oropharyngeal, and anal), saliva, and sputum. The virus can also be found in other bodily fluids like blood, urine, and feces. This is due to the high expression of ACE2 receptors in the cells of the blood vessels, kidneys, and intestines (Salamanna et al., 2020). In addition, the expression of transmembrane protease, serine 2 (TMPRSS2), an enzyme that cleaves the S protein of SARS-CoV-2 and substantially accelerates viral entry, is expressed in the respiratory system, gastrointestinal tract, kidneys, and bladder (Al-Kuraishy et al., 2021). Specimen collection is a crucial procedure to screen for COVID-19 infection among the population. Screenings are performed in hospitals and in distributed testing centers to allow expanded testing. Such testing is essential to limit the spread of disease, especially from asymptomatic individuals, and to protect healthy individuals from catching the infection, which is why specimen collection must be accurate, fast, and accessible to large populations.

### 3.1 Swabs

#### 3.1.1 Nasopharyngeal swabs

Nasopharyngeal (NP) swabs are long Q-tips inserted in one nostril through the nasal passage to collect specimens for the diagnosis of infectious diseases. This technique is the most widely used with a sensitivity that reaches 98 % (Wang et al., 2020, CDC, 2022). It is considered the gold standard to collect SARS-CoV-2 samples safely and conveniently as there is no direct contact between the health practitioner and the specimen (Marty et al., 2020). In this procedure, patients are asked to tilt their heads up to insert the swap horizontally through the nasopharyngeal passage. The swab is then kept for a few seconds to absorb enough specimens. After that, the swab is gently removed while rotating, and the tip is placed inside a tube. The applicator is then discarded by cutting it off to isolate the tip (Hiebert et al., 2021). However, this technique is found to be painful and leads to inconvenience and discomfort for patients (Butler-Laporte et al., 2021). In addition, it requires technical expertise, and healthcare providers must be protected with personal protective equipment (PPE) to obtain the sample since close contact with the patient is required.

#### 3.1.2 Oropharyngeal swabs

In addition to the NP swabs, oropharyngeal (OP) swabs target the oral cavity and reach the posterior wall of the oropharynx to collect the specimen. The tip is rotated a few times, then removed and inserted in a tube; the applicator is discarded afterward to isolate the cotton piece. This procedure is generally easier and less painful for patients; however, it has less sensitivity than NP swabs (Wang et al., 2020, Khiabani and Amirzade-Iranaq, 2021), and the practitioner must avoid contacting the swab with the tongue, palate, or uvula while collecting the specimen. Hence, using a tongue depressor may help guide the swab along the passageway (Coden et al., 2020). As with NP swabs, obtaining OP swabs requires close contact with patients, thus, using PPE is crucial.

#### 3.1.3 Anal swab

Few studies used anal swabs to diagnose COVID-19 (Abdullah et al., 2021, Li et al., 2021). However, it is not recommended to be used as a standalone test. Also, these swabs cause discomfort for the patient, and researchers are not sure if these swabs are necessary for population testing (DeutchWelle, 2021). Further research has proved that SARS-CoV-2 RNA is present in feces (Li et al., 2021, Lin et al., 2020b, Ling et al., 2020, Xiao et al., 2020) because the virus can pass through the gastrointestinal system and target ACE2 receptors that are abundant in epithelial cells of both lungs and intestines. The virus can remain in the patient’s fecal samples for weeks after recovery; hence, these specimens can determine whether the person was previously infected with the virus (Xiao et al., 2020).

### 3.2 Saliva

Unlike NP and OP swabs, acquiring saliva specimens is non-invasive, and patients will not experience discomfort. Samples can be collected by patients themselves by pouring their saliva into a container and handing it to a health provider. Saliva testing is simple, inexpensive, and accessible as it allows for rapid pooled sample testing (Tan et al., 2021, Wei et al., 2021b). Even though there is no direct contact with patients, saliva specimens encompass higher infection risks when handled by health providers. It was found that saliva specimens encompass more RNA copies per milliliter than NP swabs, with a slightly higher sensitivity in detecting SARS-CoV-2. NP swabs or saliva specimens still contain SARS-CoV-2 RNA in patients who show no symptoms (Aoki et al., 2021). However, the sensitivity of this procedure can be affected by the collection process and the homogeneity of the specimen—either pure saliva or mixed with mucus or sputum. A study showed 73.1 % and 97.6 % sensitivity and specificity, respectively, when collecting saliva samples from patients in screening centers and used the gold standard as a reference (Senok et al., 2020); while another study reported a sensitivity that reached 91 %, indicating saliva as reliable specimens to diagnose COVID-19. Since this technique is non-invasive, it allows easier opportunity to test children.

### 3.3 Other body-liquids/tissues

SARS-CoV-2 RNA can be detected in other types of specimens, including sputum, collected from the lower respiratory tract of patients who experience severe coughing. It had a considerably higher sensitivity reaching 91.6 %, providing a better indication of viral load that persists longer (Khiabani and Amirzade-Iranaq, 2021, Ravi et al., 2020; Yu et al., 2020). In addition, blood samples contain the viral RNA in lower loads (Li et al., 2021, Ling et al., 2020, Xiao et al., 2020), while most reports did not detect it in urine samples, as surveyed by Walsh et al. (Walsh et al., 2020). Feces, on the other hand, were reported to have a higher detection rate (53.42 %) since ACE2 and TMPRSS2 are abundantly expressed in the gastrointestinal tract (Salamanna et al., 2020, Al-Kuraishy et al., 2021, Xiao et al., 2020). These additional samples can be used for confirmation tests or further study the virus’s infection pathway, rather than a standalone sample for diagnosis.

The abovementioned specimen types are variable for their diagnostic accuracy; sample collection or handling, the amount of viral load in the specimen, severity, or time from the onset of infection are all factors that would affect the accuracy of diagnosis (Coden et al., 2020, Abdullah et al., 2021, Ling et al., 2020, Senok et al., 2020). Some researchers noticed variabilities when testing different specimens from the same patient (Li et al., 2021, Xiao et al., 2020, Senok et al., 2020). SARS-CoV-2 resides in the lungs (lower respiratory tract) in the early disease onset, not in the upper respiratory tract; hence, samples taken from this tract might show negative results (Li et al., 2021). Specimens with higher viral loads and sensitivities, like NP swabs and sputum, must be considered to prevent exposing healthy individuals to the infection because of false-negative results. Literature suggests collecting double patient samples to validate or confirm the results (Khiabani and Amirzade-Iranaq, 2021).

## 4 Detection methods of SARS-CoV-2

The current methods commonly used in SARS-CoV-2 detection and their properties such as sensitivity, the limit of detection (LOD), detection time, advantages, and limitations are summarized in Table 2. These methods are divided into five 1) molecular-based (RT-qPCR, ddPCR, and RT-LAMP), 2) sequencing-based, and 3) serological methods (ELISA, LFA, CLIA, and neutralization assay), 4) CRISPR technology, and 5) Biosensor-based. The critical points of each assay are described in the following sections.

**Table 2:** Summary of the detection methods and their properties*.*

Method	Type(s) of specimens	Sensitivity (%)	LOD	Detection time	Advantages	Limitations	References
RT-qPCR	NP and OP swabs, sputum.	95–100	100–500 copies/reaction	4 h	High sensitivity and specificity for SARS-CoV-2 detection (gold standard).	Requires expensive equipment and trained personnel. Gives false results in samples with low viral loads.	Behera et al., 2021, Kudo et al., 2020
ddPCR	NP swab, sputum.	94	11.1–123.2 copies/reaction	5 h	Can accurately detect the virus in samples with low viral load, reducing false-negative results.	Expensive and time consuming.	Yu et al., 2020, Suo et al., 2020
RT-LAMP	NP and OP swabs, saliva.	93.5–97.5	100–200 copies/reaction	30 min	Low cost, rapid, and highly specific.	Sensitivity depends on the viral load; some samples give intermediate results.	Aoki et al., 2021, Oliveira et al., 2021, Thi et al., 2020.
Sequencing-based methods	NP swab	99	4.08 ng/μl	24 h	Can determine the virus origin and mutations.	Expensive. Not suitable for large-scale testing. Sequencing errors occur due to a large number of reads or low viral loads in clinical samples.	Harilal et al., 2020, Shaibu et al., 2021, Slatko et al., 2018
ELISA	Blood/serum.	80–85.7	1.953–500 ng/mL	5 h	Can detect recent or previous exposure to SARS-CoV-2. Determines potential serum donors for critically ill patients.	A long time is required to develop assays. Does not directly indicate the presence of infection. Results depend on an individual’s immunity.	Carter et al., 2020, Iruretagoyena et al., 2021, Vernet et al., 2021
LFA	NP swab, saliva.	84	0.65 ng/mL	15–30 min	Rapid, small size. Does not require specialized equipment.	Gives false-negative results in samples with low viral load. Needs optimization.	Li et al., 2020, Grant et al., 2020
CLIA	Blood/serum.	73.3 for IgM, 76.7 for IgG	10 AU/mL	40 min	Rapid. Consumes low amounts of reagents.	Expensive. Results’ accuracy varies based on the time from the disease onset.	Cinquanta et al., 2017, Infantino et al., 2020.
Neutralization assays	Human epithelial cells	95–100		3–5 days	Crucial for vaccines development.	Tests must be performed in level 3 biosafety cabinets.	Behera et al., 2021, Carter et al., 2020, Abe et al., 2020
CRISPR technology	NP swab.	80–97.1	10–100 copies/reaction	30–60 min	Rapid and simple. Does not require expensive equipment.	Viral mutations cause false results.	Bokelmann et al., 2021, Broughton et al., 2020.
Biosensors	NP swab, sputum.	99	1–10 copies/reaction	10 min	Rapid, cost-effective. Most biosensors are label-free. Provide real-time measurement.	Produce small response when using small analyte quantity.	Carter et al., 2020, Abid et al., 2021, Chaibun et al., 2021
Nano-based sensors	NP swab.	100	0.18 ng/µl	20–60 min	Highly sensitive and robust. Simple. Low analyte quantity is sufficient. Improve detection accuracy.	Expensive Require further clinical experimentation.	Gupta et al., 2020, Patra et al., 2020, Zhu et al., 2020.

Abbreviations: RT-qPCR, reverse-transcription polymerase chain reaction; ddPCR, droplet digital PCR; RT-LAMP: reverse-transcription loop-mediated isothermal amplification; ELISA, enzyme-link immunosorbent assay; LFA, lateral flow assay; CLIA, chemiluminescent immunoassay; NP, nasopharyngeal; OP, oropharyngeal; IgG, immunoglobulin G; IgM, immunoglobulin M; CRISPR, clustered regularly interspaced short palindromic repeats.

### 4.1 Molecular-based methods

#### 4.1.1 RT-qPCR

Since its emergence, the reverse transcription quantitative polymerase chain reaction (RT-qPCR) was the first approved standard technique to detect SARS-CoV-2. RT-qPCR is a molecular diagnostic technique that detects the presence of nucleic acids by targeting and amplifying specific genes, resulting in creating millions of copies from a small number of nucleic acids that can be monitored in real-time (Aoki et al., 2021, Alvarez and Nourbakhsh, 2014, Tombuloglu et al., 2021). Primers are one of the key components in this reaction as they bind to the targeted gene in the DNA strand. They must be well designed with high specificity to avoid non-specific amplification and unwanted structures such as primer dimer, yielding false-positive results. DNA probes or fluorescent dyes like SYBR green indicate the presence of the targeted gene. SYBR green is an intercalating dye that binds to all double-stranded DNA (dsDNA) and produces a fluorescent signal. Probes, on the other hand, consist of a fluorescent reporter that binds to the 5′-end and a quencher dye that binds to the 3′-end on a specific target in the DNA. Once the enzyme DNA polymerase cleaves the probe, the released reporter emits a fluorescent signal, which the instrument detects (Alvarez and Nourbakhsh, 2014).

**Fig. 2:**
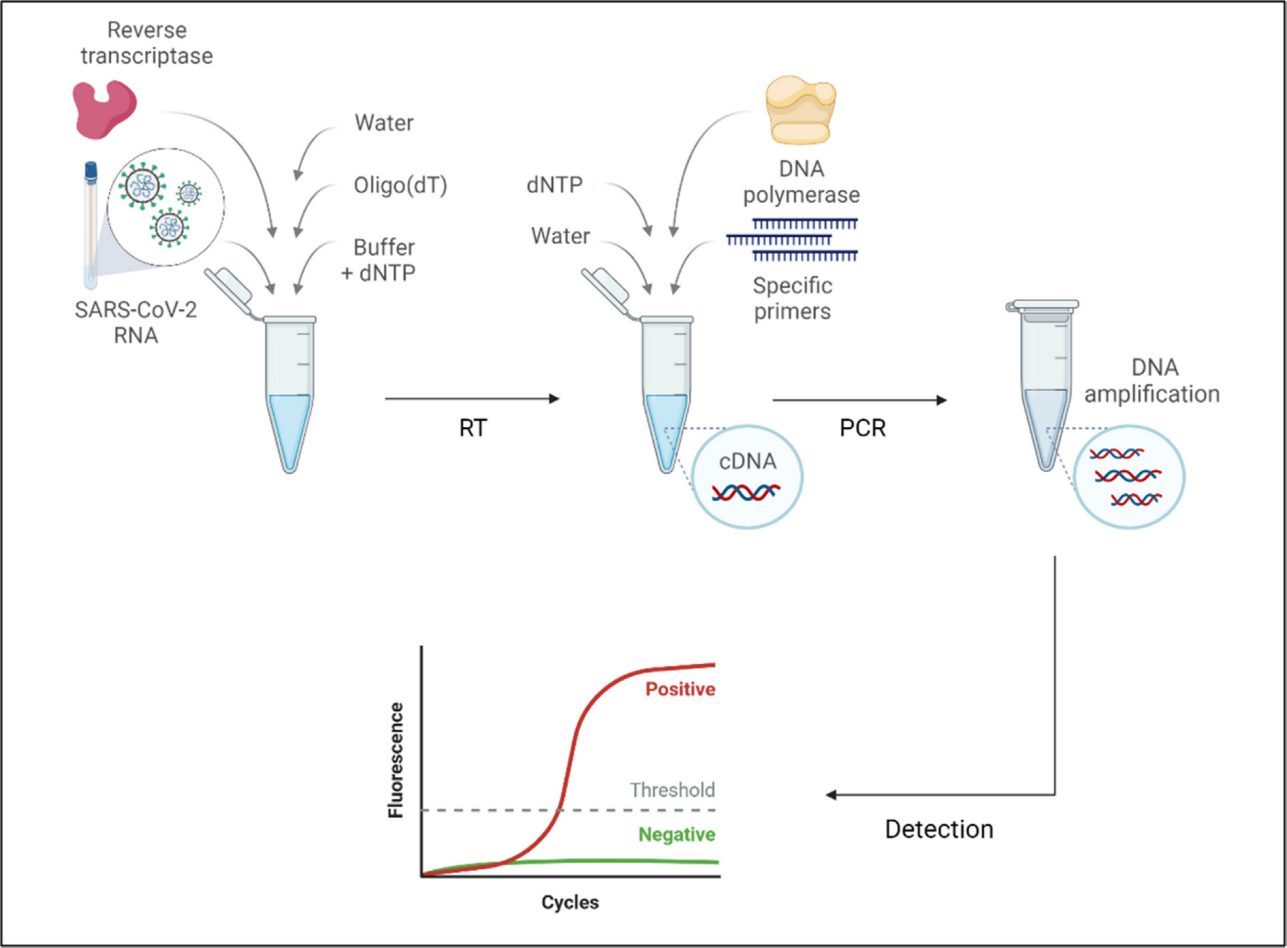
Reverse transcription quantitative polymerase chain reaction (RT-qPCR) basic steps. Complementary DNA (cDNA) is first synthesized by preparing a master mix containing an RNA template and reverse transcriptase enzyme. Then, another master mix that includes gene-specific primers and the enzyme DNA polymerase is added to initiate the PCR reaction, resulting in millions of DNA targeted sequence copies. Real-time fluorescence detection shows the amplification curve for positive samples.

Before the reaction, the viral RNA is isolated from the collected specimen using commercially available RNA extraction kits. This is crucial as proper sample handling and avoiding contaminants assure accurate results. Next, complementary DNA (cDNA) is synthesized using a master mix containing the reverse transcriptase enzyme. The mixture is then incubated in a thermal cycler to initiate the reaction. After that, cDNA, primers, distilled water, and DNA probe or SYBR green are pipetted in a 96-well plate and placed in the RT-qPCR instrument with the appropriate thermal cycles’ settings (Fig. 2). A negative control must be used to confirm the absence of contaminants and to avoid interpreting false-positive results, which is necessary for diagnosing COVID-19 patients. A positive control should also be used to avoid interpreting false-positive results. Generally, the amplification is achieved through three basic steps: denaturation, in which the temperature increases to separate the dsDNA; annealing, where the temperature drops to allow primers to bind to the targeted sequences; and extension, in which the temperature rises, allowing DNA polymerase to perform primer extension by adding nucleotides to the new resulted DNA strand. This procedure is repeated about 40 times, and new copies of DNA will result after each cycle. A fluorescent signal will be produced each time the probes are released, or SYBR green binds to the newly made dsDNA. Hence, the fluorescent signal intensity increases with the increased DNA copies after each cycle. Ct values, or cycle threshold, indicate the number of the required cycles for the fluorescent signal intensity to exceed a predefined threshold. Samples with greater viral load will have a lower Ct value; in contrast, higher Ct values indicate small amounts of RNA copies in a given sample (Alvarez and Nourbakhsh, 2014, Bustin and Nolan, 2020).

RT-qPCR is the gold standard for diagnosing COVID-19 because it is rapid, accurate, and specific in detecting and amplifying SARS-CoV-2 RNA (Bustin and Nolan, 2020). Highly sensitive primers and probes are already developed in different countries to target genes like *E*, *RdRp*, and *N* (D'Cruz et al., 2020, Tombuloglu et al., 2022). Many researchers use similar approaches when detecting SARS-CoV-2, and many are trying to overcome some limitations. For instance, Kudo et al. (2020) used a one-step multiplex RT-qPCR with primer-probe sets that target *N1* and *N2* genes, as advised by the U.S. CDC, to detect SARS-CoV-2 from saliva and NP swabs of 59 diseased and healthy individuals. They included human *RNase P* (*RP*) as an internal control in the reaction. They found that this technique was highly efficient and sensitive as it could detect low viral RNA copies, concluding that it saves time, reagents, and cost (Kudo et al., 2020). Also, others used primers that target the *E* gene and *RP* gene as an internal control along with nuclease-free water for non-template control (NTC) while testing 466 healthy and infected samples. They obtained Ct values that range from 13.38 to 34.65 for positive samples, indicating the accuracy of this method and the necessity to use internal controls to validate the results (de Oliveira Coelho et al., 2021).

RT-qPCR technology is unquestionably specific and sensitive in diagnosing COVID-19, hence being the gold standard. However, this equipment is expensive and must be operated by experienced and trained users due to the potential contamination that can affect the highly sensitive RNA extracted from specimens. Also, considerable time is needed to get the results, which adds to the limitations when underdeveloped countries with limited resources need broad COVID-19 testing (Aoki et al., 2021, Oliveira et al., 2021). It was reported that RT-qPCR was either not able to detect SARS-CoV-2 in samples with low viral loads or resulted in Ct values over 35–40. As a result, these patients can be diagnosed as negative, putting them and their families at risk for infection. Furthermore, the global demand for kits, instruments, reagents, trained personnel, and even specimen-collecting tools leads to a shortage in some regions, making it necessary to call for an alternative testing method. To avoid false results, multiplex assays were found to be helpful, even in the event of viral mutations (Kudo et al., 2020). Reduced detection sensitivity was reported when testing different RT-qPCR assays on Omicron variant-positive specimens, especially those that target a mutated region (Ippoliti et al., 2022). Therefore, SARS-CoV-2 mutations must be closely screened, and the RT-qPCR assays should be optimized and updated accordingly (Chen et al., 2022, Sharma et al., 2022). Generating a standard curve using internal controls such as *RP* and a negative control assures the accuracy of results (D'Cruz et al., 2020).

#### 4.1.2 Droplet digital PCR (ddPCR)

This technique was previously developed (Hindson et al., 2011) to reduce false-negative results limitation reported in RT-qPCR because it can detect small traces of DNA. The device divides samples into thousands of water–oil emulsion droplets, each carrying a PCR reaction. This is done by placing a cartridge containing the PCR mix into a droplet generator. Droplets are then pipetted in a 96-well plate and placed in a thermal cycler. Injecting a spacer fluid afterward separates each droplet to be detected as positive or negative based on fluorescent signals. Unlike expressing results in relative Ct values as in RT-qPCR, ddPCR provides the absolute number of gene copies in a sample using Poisson statistics. In addition, studies agreed that ddPCR was more sensitive and specific in detecting SARS-CoV-2 in samples with low RNA abundance (Behera et al., 2021, Falzone et al., 2020). For instance, a study that compared the accuracy between RT-qPCR and ddPCR when targeting *ORF1ab* and *N* genes found that 26 samples that tested negative in RT-qPCR were tested positive in ddPCR (Suo et al., 2020). Similarly, Yu and colleagues targeted *ORF1ab* and *N* genes in different specimens and revealed that ddPCR performed better in detecting the virus in samples with low viral load (Yu et al., 2020). Nevertheless, ddPCR requires expensive instrumentations and takes longer than RT-qPCR to return the results (Table 2) (Behera et al., 2021, Falzone et al., 2020).

#### 4.1.3 RT-LAMP

**Fig. 3:**
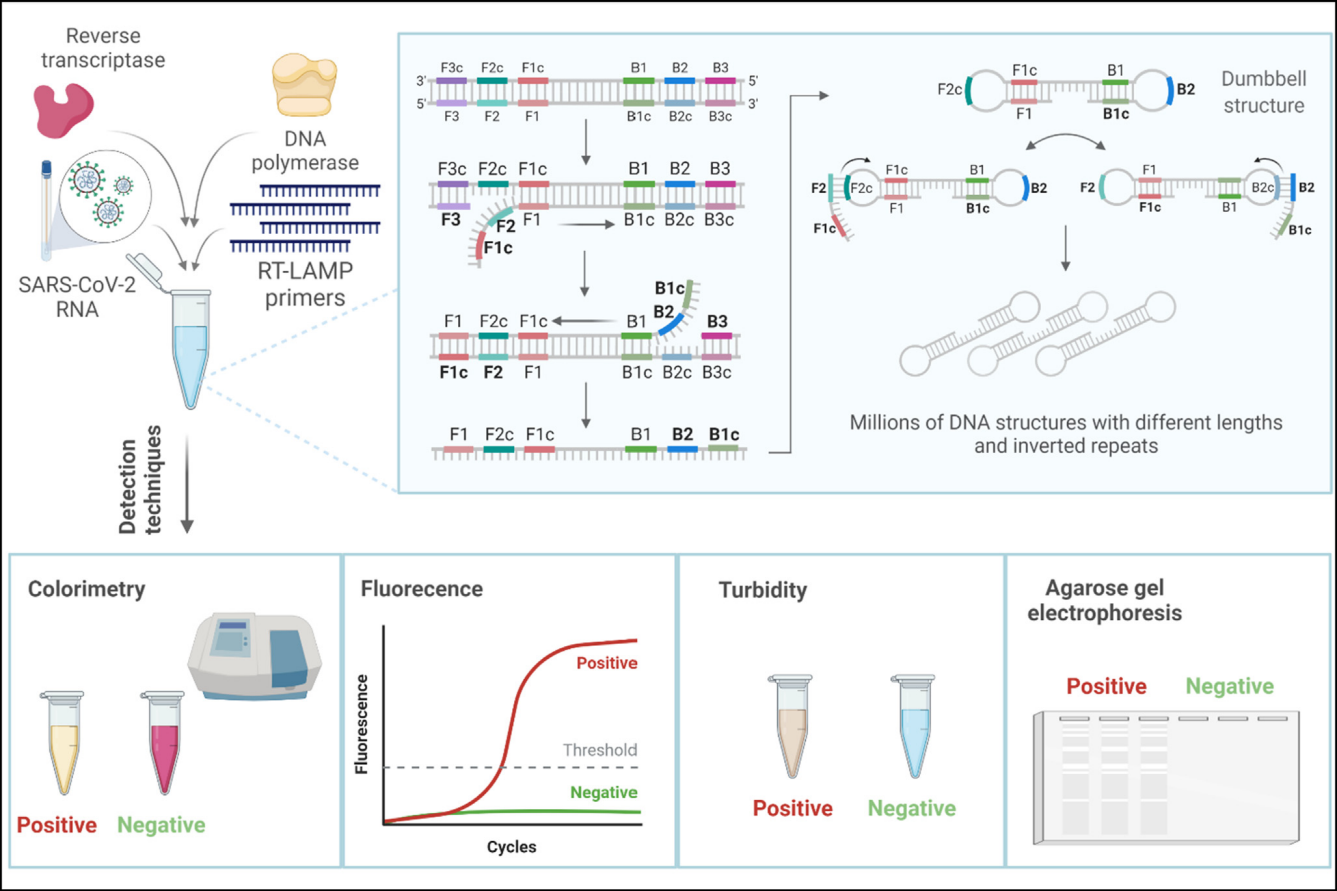
Reverse transcription loop-mediated isothermal amplification (RT-LAMP) single-tube reaction contains the RNA template, four primers, reverse transcriptase, and DNA polymerase enzymes. In this reaction, forward inner primer (FIP), forward outer primer (F3), backward inner primer (BIP), and backward outer primer (B3) bind to their complementary regions on the targeted DNA sequence (cDNA). New strands are synthesized afterward by DNA polymerase enzyme, in which complementary sequences cause the formation of dumbbell structures. Further amplifications result in millions of DNA inverted repeats with different lengths that can be detected by techniques like colorimetric, fluorescence, turbidity, and agarose gel electrophoresis.

Another molecular technique to diagnose COVID-19 that grabbed the attention of many scientists for its simplicity and high specificity is reverse-transcription loop-mediated isothermal amplification (RT-LAMP). It is a simple, quick, and highly specific technique that diagnoses viral diseases by targeting and amplifying six regions in the DNA, using 4–6 primers. They include forward and backward inner primers, and forward and backward outer primers, designated as FIP, BIP, F3, and B3, respectively, in addition to forward and backward loop primers (LF and LB) (Alhamid and Tombuloglu, 2022). Compared to RT-qPCR, RT-LAMP is a single-tube reaction that requires a single enzyme, along with reverse transcriptase, and takes place under a constant temperature setting of 60–65 °C; thus, it does not require a thermal cycler, providing a cheaper point-of-care testing alternative while maintaining sensitivity and specificity (Notomi et al., 2015). In this method, an RNA sample is isolated from the specimen and converted to cDNA by the reverse transcriptase enzyme through BIP annealing. RT-LAMP reaction starts in the same tube containing DNA polymerase, primers, and other substrates and is then placed in a 60–65 °C incubator for 15 min up to 1 h to get the results. Throughout the reaction, primer B3 anneals to its complementary region and starts complementary polymerization, displacing the cDNA strand synthesized by BIP. FIP anneals to the released cDNA strand, similarly starting displacement and polymerization. Then, the F3 primer binds outside FIP to its complementary region, and polymerization also starts there, eventually forming dumbbell structures harboring complementary sequences on both ends. After that, DNA synthesis starts at the loop regions, releasing millions of stem-loop DNA targeted sequence structures with different lengths and inverted repeats (Notomi, 2000) (Fig. 3).

RT-LAMP technique is currently used to test for COVID-19. Several reports proved its high specificity against SARS-CoV-2 (Aoki et al., 2021; Thi et al., 2020; de Oliveira Coelho et al., 2021; Bokelmann et al., 2021; Lu et al., 2020). Results from RT-LAMP must be validated by comparing them with the gold standard RT-qPCR (Thi et al., 2020). Due to the high number of primers, the possibility of forming primer dimers increases, leading to false-positive results. This is why primers must be well designed to target the desired genes specifically. A study compared 19 sets of primer assays and found that primer sets that target the *N* gene had the fastest amplification and higher sensitivity in detecting SARS-CoV-2 (Dong et al., 2021). In another study, however, other primer sets targeting the *N* gene had a Ct of about 35 and showed less sensitivity than *E* gene primers (Thi et al., 2020). As with other methodologies, using negative and positive controls will ensure accurate results. This technique offers simple detection methods including colorimetric, fluorescence, turbidity, and gel electrophoresis, as reviewed in the following sections.

#### 4.1.4 Detection methods of RT-LAMP

##### 4.1.4.1 Colorimetric RT-LAMP

One of the simplest detection methods in RT-LAMP reaction is colorimetry, which depends on the pH change as an indicator. This method is the most convenient because the results can be visible to the naked eye via a color change. Phenol red, neutral red, and cresol red dyes are among the most common indicators that are added to the reaction. Colorimetric kits are commercially available and ready to use to give results in a short time. If a positive sample is present, the pH is lowered, and the color changes depending on the dye used. This color change can be quantified by measuring the absorbance at 434 nm and 560 nm wavelengths using spectrophotometry. Many reports used this method for its simplicity, and the fact that expensive equipment like real-time fluorescence is not required makes this detection method cheap, accessible, and suitable for point-of-care testing. For example, one study used a commercially available kit to target *N* and *ORF1a* genes using colorimetric RT-LAMP, eventually resulting in a color change from red to yellow in positive samples. They postulated that those samples with Ct < 30 showed a robust color change within 30 min, while those with Ct > 30 had no color change or did so after 35 min when tested in RT-qPCR. In addition, they quantified their results by measuring the optical density (ΔOD) at 434 and 560 nm wavelengths (Thi et al., 2020). Another study detected *RdRp* and *E* genes with different respiratory viruses, including other coronaviruses, to assess the specificity, and no false-positive results were obtained; i.e., no cross-reactivity was observed. They concluded that their results had a sensitivity of 200 RNA copies with Ct < 30. The results were also quantified using spectrophotometry (Aoki et al., 2021). Others used the cresol red indicator and observed an apparent color change in samples with higher viral copies in under 40 min, while those with fewer RNA copies gave a very faint color change or required a longer reaction time for the results to occur (Lu et al., 2020).

This detection technique showed promising results, but it still has some limitations. A color change was observed in NTC when the reaction temperature exceeded 65 °C, stating that high temperatures increase the chance of forming primer dimer structures. Poor results were acquired in colorimetric RT-LAMP with low viral RNA loads and Ct > 30. The sensitivity of the colorimetric RT-LAMP method depends on the days from which symptoms of the disease appeared. It was reported that five days was the optimum time in which this technique gives the most accurate results, whereas sensitivity reduces if more than seven days since the onset of the symptoms has passed (de Oliveira Coelho et al., 2021). Some samples with Ct > 30 required over 30 min of reaction time to produce a color change. However, increasing the reaction time to over 30 min may lead to amplifications that are not specific, leading to false-positive results (Aoki et al., 2021). Both previously mentioned studies noticed indeterminate results when a lower viral load was present in the sample—i.e., an orange color was produced—those samples tested positive in RT-qPCR, indicating another limitation (Aoki et al., 2021, Oliveira et al., 2021). Using internal controls and negative controls helped to limit these problems, the importance of internal controls is to evaluate primers’ performance to prevent false-negative results, like using human RNA from MCF7 cancer cell line as a negative control (Aoki et al., 2021). In addition, adding specimens directly into the colorimetric LAMP reaction must be prevented, as they cause false-positive results due to changes in the pH (Bokelmann et al., 2021).

##### 4.1.4.2 Fluorescence RT-LAMP

The fluorescence detection method measures the intensity of the fluorescent signal or the amplification by adding intercalating dye like SYBR green to the samples and placing them in a fluorescent detector or qPCR instrument. Bokelmann et al. detected both *N* and *Orf1a* genes using fluorescence RT-LAMP in < 30 min and used SYBR green dye to induce a color change (Bokelmann et al., 2021). Another study used fluorescence assays to detect the *N* gene from 157 NP swabs, in which positive samples produced a sigmoid-shaped fluorescent signal and yielded 87 % sensitivity. Also, the specificity of RT-LAMP against SARS-CoV-2 was tested with other common respiratory viruses through fluorescence detection. After 50 min, only SARS-CoV-2 samples resulted in fluorescent signal amplification, while others did not, concluding that this technique is highly selective for this virus (Lu et al., 2020).

##### 4.1.4.3 Turbidity

Using a real-time LAMP turbidimeter, this method measures the precipitation of magnesium pyrophosphate, a byproduct resulting from DNA synthesis. Magnesium pyrophosphate is, therefore, an indicator (Notomi et al., 2015). The sensitivity of this detection technique was reported to be 20–200 copies when using primer sets that target *orf1ab* and *S* genes in 60 min. The change in turbidity can be observed via the naked eye or quantified by a real-time turbidimeter by measuring ΔOD at 650 nm, where the positive samples have turbidity values > 0.1 (Yan et al., 2020).

##### 4.1.4.4 Agarose gel electrophoresis

Agarose (2 %; w/v) prepared with ethidium bromide dye is widely used. Samples are typically loaded into the gel, and the positive ones appear as DNA bands that can be observed under a UV *trans*-illuminator. This method detects the presence of DNA after RT-LAMP reaction (Notomi, 2000) and confirms or validates the results obtained from other detection techniques (Thi et al., 2020). For instance, de Oliveira Coelho et al. (2021) validated their colorimetric RT-LAMP results by loading their samples into the gel. Samples that showed a color change from pink to yellow appeared as band patterns under UV light. The accuracy of the detection increases if more RNA copies are present in the specimen (Aoki et al., 2021).

### 4.2 Sequencing-based methods

Scientists used whole-genome sequencing methods to determine the origin of SARS-CoV-2, distinguish it from other respiratory pathogens, and identify the emerging variants that occurred in several countries via multiple sequence alignments. Whole genome sequencing is the first method utilized to detect and identify mutations in the emerging VOC, including Omicron. Currently, there are over 12 million whole genome sequences of SARS-CoV-2 variants submitted in the Global Initiative on Sharing All Influenza Data (GISAID) EpiCoV database by researchers worldwide. Next-generation sequencing refers to more recent techniques that are cheaper and less time-consuming than the standard Sanger sequencing because individual DNA fragments’ sequencing is done parallelly using small volumes on small panels. The principles of next-generation sequencing were previously overviewed (Slatko et al., 2018, Dorado et al., 2019). Many researchers have been sequencing the SARS-CoV-2 RNA obtained from patient samples (after retrotranscription into DNA) to study the virus further and develop therapeutic options or vaccines. For instance, Ren et al. identified *S*, *N*, and *RdRp* genes in five patients’ samples using next-generation sequencing. They distinguished the virus from other respiratory viruses and validated their results with the standard gold technique. They found 79 % and 51.8 % sequence identity with SARS-CoV and MERS-CoV, respectively, and concluded that the virus is a bat-origin CoV after performing the polygenic analysis (Ren et al., 2020). Another study identified *S* gene mutations associated with increased spread by sequencing the whole viral genome (Tegally et al., 2020). These studies showed the robustness of sequencing the SARS-CoV-2 genome for surveillance. These techniques, however, are time-consuming and expensive because they require special instruments and hence are not suitable for point-of-care testing. In addition, they can give inaccurate results when detecting long sequences due to a large number of cycles and data generated in the software (Slatko et al., 2018).

### 4.3 Serological methods

**Fig. 4:**
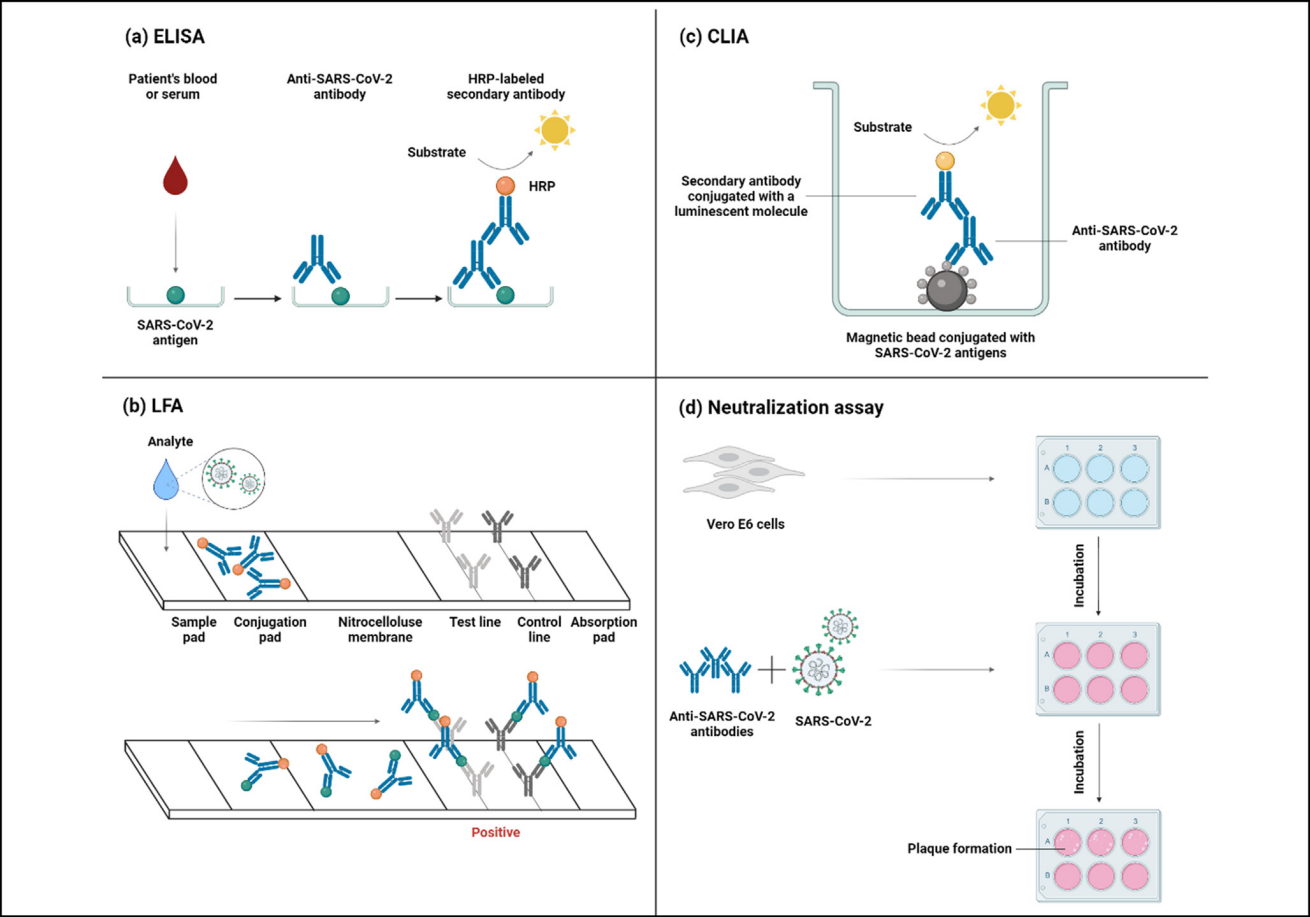
Serological methods to detect SARS-CoV-2 or anti-SARS-CoV-2 antibodies from patients' samples. **(a)** Enzyme-linked immunosorbent assay (ELISA) utilizes SARS-CoV-2 antigen immobilized on wells, and antibodies from a blood or serum sample will form antigen–antibody complexes. After washing unbound antibodies, a secondary antibody labeled with horseradish peroxidase (HRP) is added with its substrate to produce a color resulting from binding to the primary antibody. **(b)** Lateral flow assay (LFA) detects SARS-CoV-2 antigens in infected individuals by running specimens through the sample pad to the conjugation pad, where antigens bind to specific and non-specific conjugated antibodies. The complexes flow through the nitrocellulose membrane to the test line to bind to anti-SARS-CoV-2 antibodies and produce color, indicating a positive sample. The control line producing a color designates successful analyte flow. **(c)** Chemiluminescence immunoassay (CLIA) has SARS-CoV-2 antigen-conjugated magnetic beads immobilized on its surface. anti-SARS-CoV-2 antibody from a blood or serum sample binds to the antigen, which in turn, a secondary antibody conjugated with a luminescent molecule binds to the primary antibody. Eventually, a substrate is added to yield light production. **(d)** In neutralization assays, anti-SARS-CoV-2 antibodies and SARS-CoV-2 are added to the Vero E6 cell culture. This assay tests the antibodies’ ability to block the binding of the virus to cell receptors, thereby preventing plaque formation.

Antibody tests are widely used nowadays to determine whether a patient was previously infected or immune from COVID-19, as they determine the presence of antibodies or antigens in the serum, namely, immunoglobulin G (IgG) and immunoglobulin M (IgM). IgG is the most abundant neutralizing antibody and persistently remains in the blood from two weeks after the disease onset up to four months, while IgM is produced earlier and declines faster. On the other hand, IgA can be detected during the early stage of infection (within five days) and persists longer (Tantuoyir and Rezaei, 2021, Padoan et al., 2020). However, these tests do not determine if patients are currently infected because the results will return either false negative when their immune system did not generate antibodies yet, or false positive in patients who have already recovered from COVID-19 but whose blood still generates antibodies. These methods have a high specificity for SARS-CoV-2 that ranges from 96.93 to 99.91 % compared to the gold standard. As for the sensitivity, a suggestion for combining IgG and IgM seems to be the best choice rather than detecting one without the other (Vengesai et al., 2020). Most assays target the most abundant viral proteins S and N to test for antibodies (Tantuoyir and Rezaei, 2021), and other assays are coated with antibodies to target a specific antigen, the latter diagnoses COVID-19. Serological assays are plenty, but those reported in anti-SARS-CoV-2 antibodies or viral antigen detection include the following (Fig. 4):

#### 4.3.1 Enzyme-linked immunosorbent assay

Enzyme-linked immunosorbent assay (ELISA) is the most popular serological technique to detect the presence of antigens, antibodies, and proteins in clinical samples. It is widely used in laboratories for antibody testing against SARS-CoV-2 recent exposure or immunity. Some commercially available assays include recombinant SARS-CoV-2 N and S proteins targeting IgG and anti-SARS-CoV-2 IgG using S1. The first assay’s sensitivity and specificity were reported to be 85.7 % and 98.5 %, respectively, while 80 % sensitivity and 100 % specificity were reported for the latter. The samples were tested after three weeks from the onset of the disease, symptomatic patients produced higher levels of IgG compared to asymptomatic ones, and IgG titer decreased drastically in both groups after 140 days (Iruretagoyena et al., 2021). The patient’s whole blood or serum is added to a viral-protein-coated plate in this test. If antibodies are present, they will bind to antigens and form complexes, and those unbound antibodies will be washed off. Then, enzyme-labeled with horseradish peroxidase antibodies are added and bound to the antigen–antibody complex; the plates are washed again afterward. Lastly, adding a substrate will result in an enzymatic reaction that produces color. This color change indicates a positive sample and can be detected by a plate reader (Behera et al., 2021). Others developed a reproducible protocol for an ELISA assay coated with SARS-CoV-2 S protein that can detect and quantify IgG antibodies (Vernet et al., 2021). Similarly, to detect viral antigens in patient samples, ELISA plates are coated with antibodies specific to SARS-CoV-2 viral proteins. If a person is infected with COVID-19, viral antigens will bind to the coated antibodies, forming antigen–antibody complexes. Enzyme-labeled secondary antibodies and a substrate are then added to bind to the antigen–antibody complex, producing a color. This test is known as sandwich ELISA (Carter et al., 2020).

#### 4.3.2 Lateral flow assay

Besides ELISA, the lateral flow assay (LFA) detects SARS-CoV-2 antigens. It is a qualitative test that uses patients’ blood, swabs, or saliva to detect antibodies or viral antigens. This detection technique is based on the immunochromatography principle; a liquid sample is loaded on a sample pad and flows to a conjugate pad that contains lyophilized (frozen and dried) reagents, including non-specific labeled antibodies that bind to antigens. Then, antigen–antibody complexes flow through a nitrocellulose membrane into the test line and are caught by antigen-specific antibodies. Once they bind, a visible color is produced. A control test line is also present to catch the non-specific labeled antibodies to ensure that the sample has successfully flown through the test pads (Abduljalil, 2020). This assay is simple and does not require laboratory equipment, and is, therefore, suitable for rapid, point-of-care testing, as it takes 15–30 min to get the results. A study developed a small LFA strip to detect the SARS-CoV-2 N antigen in patients’ samples using specific antibodies immobilized on the strip surface; their assay had a low detection limit of 0.65 ng/mL (Grant et al., 2020). Also, LFAs are used to detect antibodies in clinical samples and developed to detect both anti-SARS-CoV-2-IgG and anti-SARS-CoV-2-IgM in two separate test lines that bind to a conjugated SARS-CoV-2 surface antigen, in addition to a control test line. In this study, they used whole blood samples loaded to the sample pad and then moved to the conjugation pad, which contained SARS-CoV-2 antigen conjugated with gold. After that, the formed antigen–antibody complexes moved through the nitrocellulose membrane containing anti-human antibodies. Once antigen–antibody complexes bind to anti-human antibodies, a color is produced, indicating the presence of IgG or IgM. They reported 88.66 % sensitivity and 90.63 % specificity (Li et al., 2020).

##### 4.3.2.1 Chemiluminescence immunoassays

Chemiluminescence immunoassay (CLIA) depends on energy release as light in a chemical-immunological reaction. A luminescent molecule is an indicator that emits a visible light signal due to electrons transitioning to the ground state after the reaction. There are direct detection methods that use luminophore molecules as a marker or indirect methods that use enzymatic markers with their associated substrates. These markers produce luminescent signals in relative light units that quantify antibodies in a sample (Cinquanta et al., 2017). These assays detect anti-SARS-CoV-2 IgG and IgM from patients’ sera. The samples are incubated in plates containing magnetic beads coupled with N antigens to form antigen–antibody complexes. Then, enzyme-labeled anti-human antibodies are added, and the plates are incubated to allow binding. Those unbound antibodies are washed off. Finally, a luminescent substrate is added to produce light that indicates the number of antibodies in the samples by a photomultiplier. The assay was reported to have 84.81 % sensitivity and 91.25 % sensitivity, requiring only 23 min to get the results (Lin et al., 2020a). Others used a similar approach and reported high sensitivity and specificity (Lu et al., 2020). Another study detected IgG and IgM antibodies in COVID-19 patients using multiplex electrochemiluminescent immunoassays coated with SARS-CoV-2 S antigen, in addition to other coronaviruses, to assess this technique’s specificity. A more robust antibody response was observed against SARS-CoV-2 compared to other coronaviruses, indicating high specificity (Infantino et al., 2020).

##### 4.3.2.2 Neutralization assays

These assays test the ability of antibodies to block SARS-CoV-2 S protein’s RBD from interacting with ACE2 receptors in host cells, thereby inhibiting viral infection and replication. In this test, a diluted patient’s serum or plasma is mixed with fully-functional SARS-CoV-2, incubated, and added to Vero E6 cell cultures. After incubation, the plates are stained, left for a few minutes, and then washed with ethanol. Antibodies binding to the viral RBD are quantified by the reduction in plaque formation in cell cultures by 50 % and 90 % compared to control (Abe et al., 2020). This technique is time-consuming as it requires days to obtain the results, and due to the high infection risk, it must be performed in a level 3 biosafety cabinet. Nevertheless, this technique is crucial in vaccine development (Carter et al., 2020). A study developed an assay similar to ELISA but used the S protein binding domain as an antigen and human ACE2 along with its enzyme and substrate as an indicator. ACE2 binds directly to RBD if antibodies fail to block its interaction. Receptor-binding domain-ACE2 interactions are detected and measured at 450 nm. The authors stated that this technique dramatically saves time, as they obtained their results in about 4 h, compared with viral neutralization assays (Abe et al., 2020).

Unlike molecular detection techniques, serological methods can detect the presence of the virus, even in samples with lower viral loads or in asymptomatic patients. As with other tests, false-negative results are expected when testing for antibodies, probably due to lower antibody levels being undetectable according to the detection threshold; hence the results depend on individuals’ immunity (Li et al., 2020).

### 4.4 CRISPR technology

CRISPR, or clustered, regularly interspaced, short, palindromic, repeats is a DNA sequence discovered in prokaryotes’ genome and is used as a defense mechanism against bacteriophages. A CRISPR-associated (Cas) enzyme is an endonuclease that cleaves the targeted nucleic acid sequence with the guide of an RNA molecule called gRNA. After its discovery, this technology was intensively invested in genome editing for therapeutic purposes. Also, CRISPR technology can detect SARS-CoV-2 gene sequences simply and accurately. The most common endonuclease enzymes used to target SARS-CoV-2 genes include Cas9, Cas12, and Cas13 (Behera et al., 2021). One of the commercially available techniques is SHERLOCK, which stands for specific high sensitivity enzymatic reporter unlocking, developed by Sherlock Biosciences. This technique combines isothermal reverse polymerase amplification and lateral flow assays. A study detected synthetic S and Orf1ab protein fragments using Cas13 endonucleases. According to their protocol, RNA is first extracted from nasopharyngeal or oropharyngeal swabs; cDNA is synthesized; an isothermal reverse-polymerase-amplification is performed using specific primers to amplify *S* and *Orf1ab* genes at 42 °C in 25 min; then, Cas13 along with gRNA is added to target and cleave the amplified sequences. A reporter RNA is also added to the mix as an indicator for the lateral flow strip, cleavage of this reporter indicates the presence of the virus. Finally, the lateral flow strip is dipped in the solution for 2 min, and the appearance of two test lines means that the sample is positive. This test can detect as few as 10–100 sequences per microliter (Zhang et al., 2020). Another technique combines RT-LAMP and a lateral flow assay, called SARS-CoV-2 DNA endonuclease-targeted CRISPR trans reporter, or DETECTR. It uses the Cas12 enzyme to cleave *E* and *N* genes. First, they extracted RNA from clinical swab samples and performed RT-LAMP with specific primer sets to amplify the targeted genes. Then, Cas12 endonuclease, with the help of gRNA, target and cleave the amplified sequences. They tested two techniques to indicate the presence of the virus in the sample: fluorescence reading using single-stranded DNA (ssDNA) probes and lateral flow strips using reporter molecules. Both detection techniques’ results agreed (Broughton et al., 2020). Table 3 below demonstrates examples of Cas9-12–13 and their subtypes used for SARS-CoV-2 detection.

**Table 3:** Examples of CRISPR/Cas9-12–13 enzymes and their subtypes developed detecting SARS-CoV-2.

Cas types	Amplification and detection methods	Target(s)	LoD	References
Cas9	Lateral flow assay combined with RT-RPA	*E* and *ORF1ab* genes.	100 copies/ reaction	(Xiong et al., 2021)
Cas12	Combines RT-LAMP amplification and fluorescence reading using ssDNA probes followed by lateral flow assay-based detection.	*E* and *N* genes.	10 copies/ µL.	(Broughton et al., 2020)
Cas12a	RT-qPCR followed by a fluorescent detection using probe reporter.	S and ORF8 proteins.	10 copies/ reaction.	(Liang et al., 2021)
Cas12b	RT-RPA followed by fluorescence detection.	*N* gene.	8 copies/ µL.	(Aman et al., 2021)
Cas13	Isothermal reverse polymerase amplification followed by lateral flow assay-based detection.	S and ORF1ab proteins.	10–100 sequences/ µL.	(Zhang et al., 2020)
Cas13a	Combines HCR and fluorescence detection.	*S*, *N*, and *ORF1ab* genes.	6 copies/ µL.	(Yang et al., 2021)

Abbreviations: LoD, limit of detection; Cas, CRISPR-associated enzyme; RT-LAMP, reverse-transcription loop-mediated isothermal amplification; ssDNA, single-stranded DNA; RT-qPCR, reverse-transcription quantitative polymerase chain reaction; ORF, open reading frame; RT-RPA, reverse transcription recombinase polymerase amplification; HCR, hybridization chain reaction.

CRISPR-based technology is advantageous because it is simple; fast, results can be obtained within 30 min up to 1 h; and cheap, as it does not require expensive instruments, therefore suitable for rapid point-of-care testing.

### 4.5 Biosensor-based approaches

Biosensors are devices that have been widely used in disease diagnosis by detecting nucleic acids, proteins, or biomarkers in specimens. Thus, they serve as promising candidates for detecting viruses, including SARS.CoV-2. These devices have three main components: a transduction element, a bioreceptor element, and a detection system. A transducer is a device that quantifies biochemical reactions to a measurable output signal, and its surface is made of a conductive material. Bioreceptor elements are molecules like antibodies, enzymes, or nucleic acids that are immobilized on the transducer surface, where analytes flow and bind to these elements to induce a quantifiable reaction. The analyte-bioreceptor element interaction alters the electrical signal measured by a detection system. The most widely used biosensors in viral detection applications include electrochemical and optical biosensors, described in the subsections below.

#### 4.5.1 Electrochemical biosensors

In electrochemical biosensors, chemical changes from analyte-bioreceptor binding produce an electrical charge that corresponds to analyte concentration and is detected by the detection system (Abid et al., 2021). Some researchers are shifting their focus to biosensor systems in detecting SARS-CoV-2 because they are simple and cost-effective. For instance, a biosensor was designed to detect S and N antigens in COVID-19 patients’ samples using a sandwich hybridization assay in an electrochemical biosensor, where immobilized anti-SARS-CoV-2 antibodies bind to antigens on a conductive electrode. The interaction caused changes in voltage as current flew through the solution. They found this technique was highly sensitive compared to the gold standard, and the results were obtained within 2 h (Chaibun et al., 2021). Another electrochemical biosensor detected S antigens using the field-effect transistor detection technique, which depends on varying electric current between two electrodes, and the resulting electrical charge accumulation is proportional to the concentration of the analyte. anti-SARS-CoV-2 antibodies are immobilized on a graphene transducer, and the interaction between the analyte and antibodies releases an electric current detected by the instrument. This technique is also highly sensitive for distinguishing SARS-CoV-2 from other coronaviruses (Seo et al., 2020).

#### 4.5.2 Optical biosensors

In optical biosensors, an incident light strikes a prism conjugated on a transducer; the detector measures the change in the refractive index of the reflected light induced by the biochemical reaction (Abid et al., 2021). Dai et al. developed a label-free surface plasmon resonance based on laser heterodyne feedback interferometry that detects SARS-CoV-2 S protein from patients’ samples. This sensor measures the change in the refractive index in response to antigen–antibody interactions in real-time, with a detection limit of as little as 0.08 pg/mL (Dai et al., 2022). Another optical biosensor detects IgG levels in patients’ serum samples using the photoluminescence spectroscopy principle. The sensor is fabricated with a semiconductor polymer layer in which an engineered RBD antigen is fixed, and it measures the change in the semiconductor polymer’s photoluminescence spectrum caused by antigen–antibody binding requiring a drop-sized sample volume (Bassi et al., 2022). Some examples of electrochemical and optical biosensors for SARS-CoV-2 antigen or antibody detection are demonstrated in Table 4.

**Table 4:** Examples of the developed electrochemical and optical biosensors for COVID-19 diagnosis.

Biosensor	Target	LoD	Reference
Electrochemical biosensors			
FET-based biosensor.	SARS-CoV-2 S protein from swab specimens	1 fg/mL	Seo et al., 2020
RCA-based electrochemical biosensor.	*N* and *S* genes.	1 copy/μL	Chaibun et al., 2021
mRT-LAMP coupled with a NP-based lateral flow biosensor assay	*ORF1ab* and *N* genes from swab samples.	12 copies/reaction	Zhu et al., 2020
eCoVSens.	S protein from saliva samples.	10 fM	Mahari et al., 2020
Optical biosensors			
dual-functional plasmonic biosensor.	SARS-CoV-2 gene-specific sequence.	0.22 pM	Qiu et al., 2020
Photoluminescence spectroscopy-based optical biosesnor.	IgG from serum samples.	0.0125 μg/mL	Bassi et al., 2022
Laser heterodyne feedback interferometry-based SPR biosensor.	S protein.	0.08 pg/mL	Dai et al., 2022

Abbreviations: LoD, limit of detection; FET, field effect transistor; SARS-CoV-2, severe acute respiratory coronavirus 2; RCA, rolling circle amplification; mRT-LAMP, multiplex reverse transcription loop-mediated isothermal amplification; ORF1ab, open reading frame 1ab; IgG, immunoglobulin G; IgM, immunoglobulin M; RdRp, RNA-dependent RNA polymerase; SPR, surface plasmon resonance.

Biosensors’ high rapidity, sensitivity, and specificity make them an excellent point-of-care testing alternative as they provide real-time viral detection. Electrochemical biosensors may be favored over optical biosensors because they can detect lower analyte concentrations, whereas lower analyte quantities yield smaller responses in the latter. Also, these sensors do not require bulk equipment, they can be easily miniaturized, and wearable for self-testing once validated in the near future (Abid et al., 2021).

#### 4.5.3 Nano-based sensors

Nano-based techniques utilize nanoparticles (NPs) to enhance the efficiency of detecting signals that arise from antigen-antibody binding. These approaches are pretty attractive in viral detection fields because NPs are stable and biocompatible in the reaction environment. Sensors that use NPs as transduction elements were found to be rapid, highly sensitive, and accurate mainly because NPs like gold (Au) are excellent conductors (Gupta et al., 2020). Nano-based approaches include conjugating NPs with biosensors, immunoassays, and molecular-based techniques. Nanotechnology detection approaches provide functional alternative methods to RT–qPCR for speedy and accurate viral detection. For the developing countries, Magnetic nano molecules can promote viral RNA extraction through co-precipitation and could be utilized for up to 50,000 diagnostic assays (Kevadiya et al., 2021). Luminescent semiconductor quantum dots (QDs) are one of the most common nanomaterials applied for biological applications. QDs are a typical tool to examine the S protein–ACE2 binding drives and consumption for their properties, such as small size, biocompatibility, photostability, and the surface easiness of reacting with biological molecules. Probes of the QDs can also support cell-based detection and study of other viral receptors. Also, gold nanoparticles (AuNPs) absorb electromagnetic radiation in the visible spectrum, so they are used as a quick (10 min) colorimetric method to detect the *N*-gene in SARS-CoV-2 that depends on the conjugating AuNPs with thiol-modified antisense oligonucleotides. After the recombinant S receptor bind to the ACE2 receptor, the fluorescence is reduced by AuNPs (Oh et al., 2005). Au-based nanomaterials can identify antibodies neutralization and recombinant proteins for either SARS-CoV-2 or those viruses that use S-mediated cell recognition and invasion via functionalizing their surface with S-specific aptamers (Aithal et al., 2022). Biosensor tools have been developed by adding plasmonic (Au and Ag) metal oxide nanoparticles and transistor bio- and graphene sensors to detect viral diseases (Farzin et al., 2020, Talebian et al., 2020). Graphene biosensors are applied for SARS-CoV-2 detection for their sensitivity since S antibodies can be immobilized on a graphene surface (Seo et al., 2020). Linking AuNPs and AgNPs to antibodies—after their binding to the virus antigen or its RNA—results in an identifiable signal to detect SARS-CoV-2 (Tymm et al., 2020). In recent work, a one-step optical S protein-specified nanoplasmonic resonance sensor was developed. This sensor does not need sample preparation and gives immediate detection of the virus via very specific immunoglobulins that bind to the S protein of SARS-CoV-2 on the nanosensor plate, eventually resulting in a plasmon resonance that can be detected visually (Huang et al., 2021, Yanik et al., 2010). Another recently developed detection sensor includes a double-functional plasmonic photothermal action based on the local surface plasmon resonance. The detection is carried out on Au nanoislands containing complement-specific receptors that homogenize to nucleic acids of SARS-CoV-2. The model is stimulated at two distinct wavelengths; the first is from the biosensor of plasmonic photothermal, while the second comes from the local surface plasmon resonance. This biosensor detects *RdRp*, *ORF1ab*, and *E* genes and has more accuracy in multigene sequences with a lower limit of detection (0.22 pM), which significantly decreases false-positive results (Qiu et al., 2020). *ORF1ab* and *N* genes in patients’ swab specimens using RT-LAMP, then dipped lateral flow immunoassay strip in the mixture to obtain the results only one hour from collecting the samples. They used polymer coated with streptavidin-NP dye immobilized on the conjugation pad, which yielded rapid detection with high sensitivity and specificity (Zhu et al., 2020). Further recent nanomaterial-based detection systems that utilize smart materials have been surveyed by Kevadiya et al., 2021, Singh et al., 2022.

NPs shape the future of diagnostics because they are small in size but provide a high surface-to-volume ratio, which allows using smaller analyte quantities and hence offer lower detection limits when using them as transduction materials. Also, NPs provide therapeutic options; NPs conjugated with drugs allow for targeted drug delivery specific to SARS-CoV-2. All the above-mentioned features of nano-based techniques make them suitable for point-of-care testing. However, clinical experiments in diagnosing COVID-19 using these techniques are still limited (Patra et al., 2020, Srivastava et al., 2021).

## 5 Conclusions and future perspective

Ideally, diagnostic methods should be accurate, scalable, rapid, and inexpensive to allow population-based testing. The *N* gene was the target of choice for many researchers who used molecular-based methods to detect SARS-CoV-2 due to its high stability (Kudo et al., 2020; Dao Bokelmann et al., 2021, Broughton et al., 2020, Chaibun et al., 2021, Dong et al., 2021, Grant et al., 2020, Lu et al., 2020, Zhu et al., 2020, Thi et al., 2020). Some researchers are testing the efficiency of SARS-CoV-2 detection directly from specimens without RNA isolation and found a good agreement compared to the standardized technique. Different methods like colorimetric RT-LAMP and RT-qPCR robustly detected the virus in positive clinical samples (NP swabs) without prior RNA extraction and did not obtain false results (Miranda et al., 2020, Wei et al., 2021a). One study, however, showed a reduced sensitivity in RT-LAMP results using direct saliva and nasopharyngeal swabs, stating that RNA isolation is a crucial step to efficiently detect the virus (Taki et al., 2021). Nevertheless, skipping RNA isolation reduces analysis time and solves the shortage of reagents and specialized instrumentations for rapid global testing in general and resource-limited countries in particular (Miranda et al., 2020). As most COVID-19 tests are molecular-based, more focus should shift to serological methods as they are essential for vaccine development and determining potential plasma donors. One issue is that developing assays require time during the fast-evolving pandemic.

RT-qPCR is still the most widely used nowadays, but it suffers from giving off false-negative results in samples that have low viral loads, ddPCR overcomes this limitation. Research is still ongoing to develop cost-effective approaches for COVID-19 detection, especially after the global economic crisis caused by the pandemic (Jackson et al., 2021). Nonetheless, authorities must take into account accuracy and cost to choose the proper detection technique based on the capabilities of each region; combining diagnostic methods with the gold standard certainly validates tests to avoid false results and their associated consequences.

## Declaration of Competing Interest

The authors declare that they have no known competing financial interests or personal relationships that could have appeared to influence the work reported in this paper.
